# Development and immunochemical evaluation of a novel chicken IgY antibody specific for KLK6

**DOI:** 10.1186/1752-153X-6-148

**Published:** 2012-12-05

**Authors:** Georgia Sotiropoulou, Georgios Pampalakis, Evangelia Prosnikli, Gregory P Evangelatos, Evangelia Livaniou

**Affiliations:** 1 Department of Pharmacy, University of Patras, 265 00, Rion-Patras, Greece; 2 Institute of Radioisotopes & Radiodiagnostic Products, Immunopeptide Chemistry Laboratory, National Centre for Scientific Research (NCSR) “Demokritos”, 153 10, Aghia Paraskevi-Attiki, Greece

**Keywords:** Kallikrein-related peptidase 6(KLK6), IgY, Immunoassays

## Abstract

**Background:**

Human kallikrein-related peptidase 6 (KLK6) has been implicated in various types of cancer and in neurodegenerative and demyelinating diseases including multiple sclerosis. Further, anti-KLK6 antibodies attenuated disease manifestations in the mouse model of multiple sclerosis. Availability of specific antibodies against KLK6 is fundamental to the development of improved diagnostic and/or immunotherapeutic applications. Here, we exploited the enhanced immunogenicity of mammalian proteins in avian species to generate a polyclonal antibody against KLK6.

**Results:**

Chicken were immunized with recombinant KLK6 and antibodies Y (IgYs) were purified from egg yolk with a simple procedure and evaluated for KLK6 detection by ELISA and Western blot using recombinant proteins and human cell lysates and supernatants. The anti-KLK6 Y polyclonal exhibited high affinity for KLK6 with a detection limit of 30 fmol. On the other hand, the widely used rabbit polyclonal antibody that was raised against the same recombinant KLK6 had a detection limit of 300 fmol. Moreover, the IgYs did not display any crossreactivity with recombinant KLKs or endogenous KLKs and other cellular proteins.

**Conclusions:**

Based on its high specificity and sensitivity the developed anti-KLK6 IgY is expected to aid the development of improved diagnostic tools for the detection of KLK6 in biological and clinical samples.

## Background

Y antibodies are the predominant serum immunoglobulins in birds, reptiles and amphibians. In the female, transfer of IgYs from serum to egg yolk confers passive immunity to embryos and neonates [1,2] similarly to placental IgG transfer in mammals which also confers passive immunity to the fetus. The enrichment of these immunoglobulins in egg yolk led Leslie and Clem to name this antibody class IgYs [3]. There are several advantages associated with the development and usage of Y antibodies, including better immune responsiveness to mammalian antigens, higher affinity with persistent titer, non-invasive collection, simple and low cost isolation process, large yield and scalable production [1], while enhanced immune response results in antibodies with improved specificity and sensitivity as compared to mammalian IgGs. In this study, we exploited these advantages for the generation of a novel polyclonal that recognizes the KLK6 protease with high affinity and specificity.

KLK6 was originally identified and cloned based on its aberrant expression in mammary and ovarian cancers and was proposed as a potential diagnostic biomarker [4]. Now it is known that KLK6 has a tissue-wide range of expression, including breast, central nervous system, kidney *etc.*[5]. It should be emphasized that KLK6 has many transcript and splice variants [6,7]. Transcript variants result from alternative promoters usage and encode for the same KLK6 protein, since all mRNA sequence changes occur at the 5^′^-untranslated region, while splice variants result mainly by skipping coding exons and they either encode for small proteins with low identity to KLK6 or to no proteins at all [6,7].

Recently, it was shown that stromal cell-associated expression of KLK6 is an indicator of poor prognosis in ovarian cancer patients [8]. KLK6-positive ovarian cancer patients show an increased risk of relapse and death compared to KLK6 negative [9], and the combination of KLK6 and CA-125 enhances the diagnostic power [10].

Additionally, KLK6 has been found up-regulated both in tumor specimens and serum of patients with colon cancer and high KLK6 expression was associated with poor prognosis [11]. Elevated expression of KLK6 in gastric cancers as compared to noncancerous tissues was associated with lymphatic invasion [12]. Finally, KLK6 shows strong expression in low grade in contrast to high grade renal cell carcinomas [13] and represents a potential serum biomarker for uterine serous papillary cancer [14].

The role(s) of KLK6 in the progression of human malignancies are not clear and they may vary with the type of cancer and KLK6 levels of expression [15]. KLK6 has been shown to cleave components of the extracellular matrix (ECM), therefore it was concluded that KLK6 promotes cancer invasion and metastasis [16]. In addition, KLK6 was shown to release angiostatin for plasminogen, thus it may have anti-angiogenic potential [17]. In breast cancer expression of KLK6 at physiological levels has tumor-suppressor properties, while over-expression results in tumor promotion [18]. Overexpression of KLK6 in lung cancer is related to increase in cyclin E and increase in cell proliferation [19].

KLK6 has also been suggested as a new biomarker for neurodegenerative diseases. Specifically, plasma levels of KLK6 were found reduced in patients with Alzheimer disease or other forms of dementia compared to age-related healthy individuals [20,21]. Further, KLK6 was found elevated in the serum of multiple sclerosis patients with highest levels being associated with secondary progressive disease [22]. On the other hand, KLK6 can degrade myelin. Consequently, it is considered to play important roles in the physiological demyelination and remyelination process [23,24], while its aberrant activity has been associated with pathological demyelination typical of multiple sclerosis and other demyelinating diseases. Importantly, passive immunization through administration of anti-KLK6 antibodies or active immunization through administration of recombinant rat Klk6, to induce the production of anti-KLK6 antibodies, delayed significantly the onset and attenuated the symptoms of experimental autoimmune encephalomyelitis (EAE), the mouse model for multiple sclerosis [25]. Recently, Klk6 neutralizing antibodies slowed disease progression in the TMEV (Theiler’s murine encephalomyelitis virus) mouse demyelination model [24]. Also, KLK6 was shown to be new serum prognostic marker for aneurismal subarachnoid hemorrhage. Specifically, serum KLK6 was decreased in patients relative to adult population and the lowest concentrations were correlated with worse outcome [26].

On the other hand, a large body of emerging evidence suggests that there is a functional interaction of KLKs, including KLK6, with proteases of the thrombostasis axis [27]. Potential regulatory interaction between KLK6 and proteases of the thrombostasis axis could have a large impact in various human diseases, including neurodegeneration and cancer, as discussed above [27]. Consequently, the development of new reagents for the detection of KLK6 with potential diagnostic applications is of great importance. Presently, a limited number of rabbit polyclonal or mouse monoclonal antibodies have been generated against KLK6. Often, their specificity (i.e. lack of crossreactivity with other KLKs or non-KLK proteins) has been debated. Using IgY technology, we developed a new polyclonal antibody that displayed high specificity and sensitivity for KLK6 in Western blot and ELISA immunoassays.

## Results

### Production of anti-KLK6 IgYs

**Figure 1:**
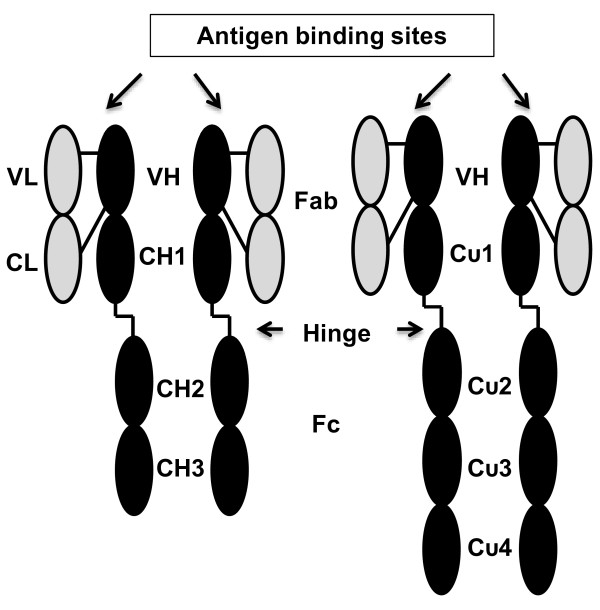
**Structure of IgYs. **Representation of Y and G antibodies [modified from 2].

Chicken were immunized with rKLK6, the laying eggs were collected and IgYs were purified by a modification of the acidified water procedure [28]. The structure of Y antibodies resembles the structure of G antibodies except for the longer Fc fragment (Figure 1) [2]. A major difference between Y and G is the inability of Y antibodies to bind to bacterial Fc receptors such as the staphylococcal Protein A or streptococcal Protein G [1].

The purity of the generated IgYs was very high and no contaminating proteins could be detected by SDS-PAGE resolution of IgYs (data not shown). The two detected bands of about ~67 kDa and 25 kDa correspond to the heavy and light chains of IgYs, respectively. The 35 kDa band of the vitellogenin II precursor observed in previous IgY preparations was absent [28]. The yield of IgY production was 100 mg per single yolk, which is substantially higher than yields for IgG production.

### Titration and displacement ELISA

**Figure 2:**
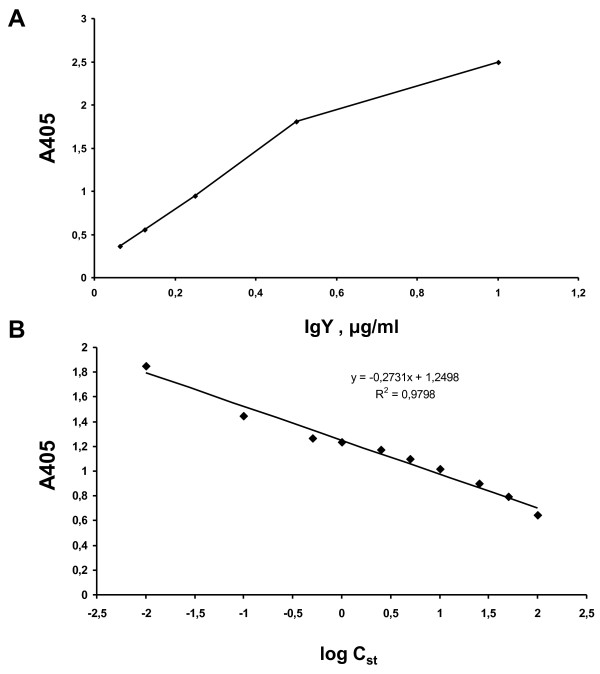
**Titration and displacement ELISAs. A**, Antibody titer determination by ELISA. **B**, Displacement ELISA.

The developed IgYs were initially evaluated by ELISA. As shown in Figure 2A, under the conditions used (details in Materials and Methods section), the titer of the obtained antibody was approximately 0.125 μg/ml (negative controls either no coating or without IgYs gave absorbance values between 0.402 and 0.509). More specifically, the 0.125 μg/ml concentration of the antibody gave absorbance values >1.5-times the negative controls. Figure 2B shows the displacement curves obtained for the antibody. The addition of rKLK6 displaced bound IgYs in a concentration-dependent manner.

### Antibody characterization

**Figure 3:**
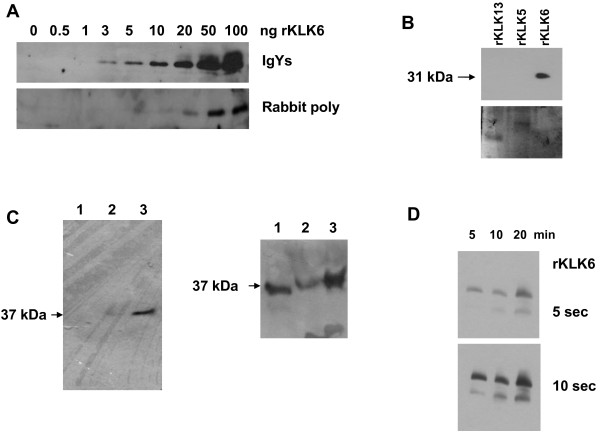
**Sensitivity and specificity of IgYs. **Recombinant and endogenous KLK6 were detected by Western blotting using IgYs at 1:2,500. **A**, IgYs could detect as low as 1 ng (30 fmol) of rKLK6, while the widely used anti-KLK6 rabbit polyclonal [30] was limited to 10 ng (300 fmol). **B**, IgYs could recognize rKLK6 (100 ng) but lacked crossreactivity with its most homologous KLK13 (rKLK13, 1.5 μg) but also KLK5 (rKLK5, 1 μg) (Western blot, upper). Integrity of the loaded proteins was confirmed by Coomassie staining (SDS-PAGE gel, lower). **C**, IgYs lack crossreactivity with any of the secreted proteins as no band could be detected in MDA-MB-231 cells that do not produce KLK6 (lane 1, left). A single band of 37 kDa corresponding to endogenous KLK6 could be detected in MDA-MB-468 and MDA-MB-231 C5WT, a cDNA-transfected clone to stably express KLK6 [18] (lanes 2 and 3 respectively; left). Moreover, lack of crossreactivity of IgYs with other cellular proteins was confirmed by analysis of whole cell lysates (100 μg) isolated from MDA-MB-231 C5WT (lane 1, right) and MDA-MB-468 (lane 3, right). Additionally, IgYs could recognize a mutant form of KLK6 produced by MDA-MB-231 C7MS transfectants (lane 2, right) that lacks enzymatic activity [18]. **D**, Heat-induced denaturation of rKLK6 increases the sensitivity of IgYs. 5, 10 and 20 min refer to times of incubation at 95°C, while 5 and 10 sec denote the time of exposure; the two bands correspond to glycosylated (31 kDa) and non-glycosylated (25 kDa) rKLK6 produced in *Pichia pastoris.*

The generated chicken anti-KLK6 antibody (IgYs) was compared with a rabbit polyclonal against KLK6 that has been widely used for analyses of biological and clinical samples [5,21,29,30]. The sensitivity of IgYs and that of the widely used rabbit polyclonal were compared in parallel by Western using serial dilutions of rKLK6. As shown in Figure 3A, the detection limit of IgYs was down to one ng or ~30 fmol of KLK6 compared to 300 fmol for the rabbit polyclonal.

Then, we tested the potential crossreactivity of the produced IgYs with other recombinant KLKs that we had produced [17]. Recombinant KLK5 and KLK13, which is the most closely related by sequence to KLK6 [31], were tested. As shown in Figure 3B, no crossreactivity could be detected even for high (1–1.5 μg) amounts of recombinant proteins. In particular, sensitivity of IgYs for detection of endogenous KLK6 and lack of crossreactivity with other KLKs and unrelated proteins was assessed by analyzing whole cell lysates and supernatants isolated from MDA-MB-468 breast cancer cell line that expresses the KLK5, KLK6 and KLK10 proteins. The MDA-MB-231 KLK6-non-expressing cell line was used as a negative control for KLK6 but a positive control for KLK1 [32]. In addition, cDNA-transfected MDA-MB-231 cells with stably reconstituted KLK6 expression were included in the analysis [18]. The IgYs could detect the endogenous (secreted and intracellular) KLK6 protein produced by MDA-MB-468 and MDA-MB-231 cells stably transfected with the KLK6 cDNA (Figure 3C) and no other proteins were detected indicating high specificity of IgYs for detection of endogenous KLK6 by Westerns. On the other hand, complete absence of crossreactivity of the anti-KLK6 IgYs with endogenous KLK5 and KLK10 or any other KLK-unrelated proteins could be demonstrated in lysates and supernatants of MDA-MB-468. Also, lack of crossreactivity with KLK1 was shown in MDA-MB-231 cells (Figure 3C). If KLK10 was detected in MDA-MB-468 it would have given a band that would not overlap with the KLK6 due to difference in molecular weight (30 kDa for KLK10 compared to 37 kDa for glycosylated KLK6). In addition, KLK6 protein was detected in differentiated SH-SY5Y neuroblastoma cells, while other bands derived from non-specific binding of IgYs could not be detected (Vekrellis, Sotiropoulou et al. unpublished data).

Finally, we showed that denaturation of the KLK6 protein due to prolonged heating at 95°C enhanced the sensitivity of IgYs against KLK6 (Figure 3D). This observation can be exploited in Westerns to reduce the detection limit of IgYs below the 1 ng (30 fmol) KLK6 threshold. The generated IgYs were shown unable to immunoprecipitate KLK6 (not shown), which is common for Y antibodies due to their shorter hinge structure that usually renders them less effective in immune-precipitations as compared to G antibodies [1].

### Effect of IgYs on KLK6 activity

**Figure 4:**
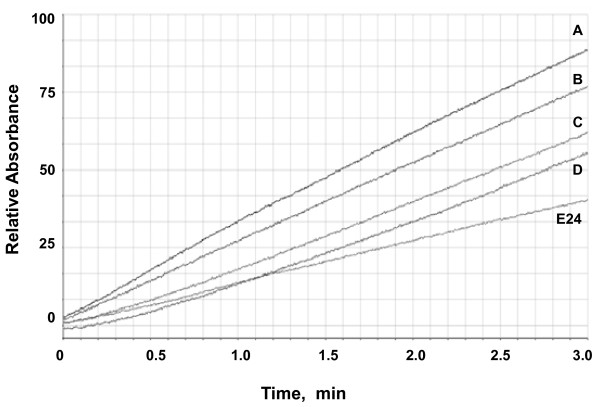
**Inhibition of KLK6 activity by IgYs. **The activity was measured in the absence (**A**) and presence of IgYs at increasing molar ratios of IgYs:rKLK6 equal to 1:1, 20:1 and 30:1 (**B**, **C** and **D**, respectively). Then, the trypsin substrate N-benzoyl-L-arginine ethyl ester (BAEE) was added and the change in absorbance at A254 was measured relative to time for 3 min. The corresponding changes in the absorbance at A254 were normalized and expressed % of the rKLK6 without addition of the antibodies. IgYs could inhibit rKLK6 only moderately as 61% of its activity remained at a molar ratio as high as 30:1, while the E24 mouse monoclonal control could extinguish 60% of the activity at 1:1. Rates of hydrolysis of the BAEE substrate by rKLK6 are shown. BAEE was used at 250 μΜ and rKLK6 at 12 nM.

IgYs were tested for inhibitory ability against the enzymatic activity of KLK6. As shown in Figure 4, partial inhibition of KLK6 proteolytic activity was observed at molar ratios IgYs:KLK6 as high as 30:1, indicative of a small fraction of IgYs recognizing three-dimensional epitopes. Thus, it may still be possible to raise antibodies with enzyme-inhibitory activity in avian species. The well-described E24 mouse monoclonal that blocks the enzymatic activity of KLK6 [16] was used as positive control, which at molar ratio 1:1 caused about 60% inhibition of KLK6 activity. Allowing KLK6-IgYs complex formation for longer time (10 min) did not increase further the extent of inhibition (not shown).

## Discussion

Despite the fact that IgYs were described about 120 year ago their applications in immunoassays have been limited compared to IgGs [1]. In the recent years however, the development of chicken antibodies is considered an advantageous alternative compared to the classical mammalian immunization procedures, mainly because mammalian proteins exhibit enhanced immunogenicity in avian species due to phylogenetic distance. Recently, the so-called IgY technology was suggested as a potential new way for passive immunization to treat human and animal diseases [33].

We applied IgY technology to high-yield generation of Y antibodies against rKLK6 that were purified to near homogeneity with a very simple and inexpensive procedure and characterized in established immunochemical methods for future validation in clinical diagnosis. The utility of our anti-KLK6 Y antibodies lies in their absence of immunological crossreactivity with enzymes of the KLK family, specifically KLK5 and KLK13 and endogenous KLK1 and KLK10, but also with any other cellular proteins produced by mammary (and neuronal) cells, likely due to the evolutionary distance between avian and human species. Lack of specificity of anti-KLK antibodies, especially their crossreactivity with multiple KLKs, has been an obstacle to the optimization of immunoassays for clinical applications. Even the extensively used and characterized anti-KLK3 (PSA) antibodies (rabbit polyclonal and mouse monoclonal antibodies) were shown to crossreact with KLK2 and KLK1 [34]. Moreover, our IgY production offers the advantages of easy scale-up to gram levels and simple purification process. Generation of 100 mg of IgGs that can be obtained from a single yolk, would require approximately 20 ml of rabbit antiserum [28].

Recently, an anti-peptide rabbit polyclonal was generated against the KLK6 (109–119) region that lacks homology with other KLKs, thus, the affinity-purified polyclonal displayed high specificity against KLK6 [8]. To the advantages of our production method, high yield and no need for affinity purification should be added. Availability of adequate amounts should facilitate pharmacological validation of IgYs for potential therapeutic applications. Indeed, anti-KLK6 therapy was effective in mouse models of multiple sclerosis [23-25]. However, our antibody has certain limitations in its applications. Specifically, we demonstrated that the developed antibody cannot be used for immunoprecipitations which is a more general drawback of Y antibodies. Further, it cannot be used as a potent inhibitor of KLK6 protease activity since it shows moderate inhibition and only in high molecular ratios.

Overall, the IgY technology will help to reduce the cost of clinical or research immunochemical tests. Further it reduces the number of animals used since a single hen can produce eggs having the desired IgYs for at least 10 months leading to the production of very high amounts of antibodies [2]. Finally, it meets the recommendations of the European Centre for the Validation of Alternative Methods (ECVAM) that specifies that IgYs are suggested to be used instead of mammalian antibodies for animal welfare purposes [35]. To our knowledge this is the first study that develops a chicken antibody against KLK6 and in general against a member of the human KLK family of serine proteases.

## Conclusions

This study generated a novel chicken polyclonal antibody against KLK6. KLK6 is an emerging new biochemical marker for clinical diagnosis of various forms of cancer, including ovarian cancer and for neurodegerative disorders. The developed antibody exhibited sensitivity in the subpicomolar range and very importantly it lacked crossreactivity with other KLK enzymes or cellular proteins.

## Methods

### Materials

All chemicals used were obtained from Sigma (St. Louis, MO, USA) or Merck (Darmstadt, Germany).

### Production of recombinant KLKs

Recombinant KLK6 (rKLK6) and other recombinant KLKs were produced in the methylotrophic yeast *Pichia pastoris* KM71 strain, purified to homogeneity and activity-tested as described [15,36,37]. The identity of the proteins was verified by N-terminal analysis by Edman degradation and/or MALDI-MS or ESI-MS, while the absence of undesired mutations was also confirmed by sequencing the cloned *KLK* cDNA fragment before yeast transformation [17,36,37]. Mature rKLK6 was used for hen immunization.

### Hen immunization

Laying hens (Leghorn hybrids) 3-month old were immunized by subcutaneous injections on the neck. The immunogen, rKLK6, was injected (100 μg per injection) as an emulsion (1:1 v/v) in Complete Freund’s Adjuvant. rKLK6 encompasses the aminoacid residues 22–244 of the Uniprot sequence Q92876, thus corresponds to the full-length KLK6 protein. Booster injections were administered every 2 weeks for a total of 3 months. Care of animals was in accordance with European legislation and our Institution’s Guidelines pertaining to the use of laboratory animals.

### Isolation of IgYs

IgYs were isolated from egg yolk according to a modified version of the acidified water dilution method [28,38]. Briefly, yolks from 15 eggs were separated from white, washed with distilled water and allowed to drip through pharmaceutical gauze into a beaker. Yolk was diluted ten times (v/v) with acidified water (pH 5.2), the mixture was remained 16 h at 4°C and, then, centrifuged at 8,500xg for 30 min at 4°C. Lipid-containing residue was discarded and supernatant was collected. Na_2_SO_4_ was added to the supernatant up to 19% concentration and the mixture remained at 37°C for 3 h and, then, at room temperature for 16 h. Subsequently, the mixture was centrifuged at 8,500xg for 30 min at 25°C and the precipitant containing IgYs (100 mg/egg determined by Bradford assay) was collected, dialyzed against water (72 h, 4°C, MWCO of 12 kDa) and lyophilized (100 mg/egg determined by Bradford assay). 3 mg of IgYs were dissolved in 1 ml PBS and stored in aliquots at −20°C.

### Cell culture and preparation of cell lysates and supernatants

All cell lines were grown as described [18]. For preparation of cell lysates, cells were lysed in 50 mM Tris–HCl, pH 8.0, 150 mM NaCl, 1% Igepal CA-630 for 30 min on ice and lysates were clarified by centrifugation at 16,000xg for 10 min and used immediately for analysis. Serum-free conditioned media (SFCM) were collected from confluent cultures at 24 h, clarified by centrifugation and concentrated by 10-fold using centrifugal filter devices (Amicon, MWCO of 10 kDa).

### Western blot

Samples were resolved on 12% SDS-PAGE and transferred onto PVDF membranes. Membranes were blocked with 5% milk in PBS. IgYs (or rabbit IgGs) was added to 1:2,500 dilution in 1% milk in PBS containing 0.05% Tween (PBST) for 1 h at room temperature. Then, membranes were washed with PBST and anti-IgY (Sigma) was added to 1:3,000 dilution in 1% milk in PBST for 1 h at room temperature. Specific immunoreactive bands were detected with West Pico ECL (Pierce). For immunoprecipitation, rKLK6 (2 μg of rKLK6 in 800 μl PBS) was pre-incubated with 20 μg of IgYs for 1 h at room temperature, then, a rabbit anti-IgY polyclonal antibody (10 μg) was added and the mixture incubated for another 1 h at room temperature. Finally, 50 μl of protein G beads (1:1) were added and incubated for 16 h at 4°C. Beads were recovered by centrifugation, proteins eluted in sample buffer and analyzed by Western.

### Titration ELISA

ELISA microtiter plates were coated with rKLK6 (100 ng/ml, 100 μl/microwell, 16 h at 37°C), then, washed and blocked with 2% BSA in PBST (200 μl/well, 1 h at room temperature). Blocking was discarded and wells were washed with PBST and incubated with serial dilutions of IgYs in PBST containing 0.2% BSA (100 μl/well, 2 h at 37°C). Following, wells were washed with PBST and incubated with rabbit anti-IgY secondary antibody coupled to horseradish peroxidase (HRP) at 1:5,000 dilution in PBST with 0.2% BSA (100 μl/well, 2 h at 37°C). Finally, wells were washed with PBST, incubated with ABTS/H_2_O_2_ (100 μl/well, 30 min at room temperature) and the absorbance was measured at 405 nm.

### Displacement ELISA

Microtiter plates were coated and blocked as described above. Then, microwells were incubated with varying concentrations of anti-KLK6 IgYs concomitantly with a series of KLK6 standard solutions. After incubation the plates were processed as described for titration ELISA.

### Enzyme kinetics

The enzymatic activity of rKLK6 [37] was measured using the N-benzoyl-L-arginine ethyl ester (BAEE) substrate in 67 mM Na_2_HPO_4_, pH 7.6. For KLK6 enzyme kinetics, rKLK6 (R80Q) was used, a mutant form of KLK6 with stabilized activity compared to wild-type that is amenable to autocatalytic inactivation *via* cleavage at R80 [37]. Rates of hydrolysis were measured based on changes in the absorbance at 254 nm monitored by a double beam UV–vis spectrophotometer (Perkin Elmer). As control for specific inhibition of KLK6 activity, the E24 anti-KLK6 blocking mouse monoclonal was used [16]. For inhibition assays, the antibodies were pre-mixed with rKLK6 and incubated for 1 min, then, substrate was added and the rate of hydrolysis was monitored.

## Abbreviations

ABTS: 2,2^′^-azino-bis-(3-ethylbenzthiazoline-6-sulfonic acid) diammonium salt; BAEE: N-benzoyl-L-arginine ethyl ester; BSA: Bovine serum albumin; IgY: Immunoglobulin Y; KLK6: Kallikrein-related peptidase 6; MWCO: Molecular weight - cut off; PBST: 0.01 M phosphate buffered saline, pH 7.4, containing 0.05%, v/v, Tween-20; SFCM: Serum-free conditioned media.

## Competing interests

The authors declare that they have no competing interests.

## Authors’ contributions

GS conceived, and designed the project, and drafted the manuscript. GP performed experiments, drafting the manuscript. EP, GE, EL performed experiments. All authors have read and approved the final manuscript.
